# How Do the Players Play? A Post-Genomic Analysis Paradigm to Understand Aquatic Ecosystem Processes

**DOI:** 10.3389/fmolb.2021.662888

**Published:** 2021-05-07

**Authors:** Thomas Reid, Jordyn Bergsveinson

**Affiliations:** ^1^ Canada Centre for Inland Waters, Environment and Climate Change Canada, Burlington, ON, Canada; ^2^ National Hydrology Research Centre, Environment and Climate Change Canada, Saskatoon, SK, Canada

**Keywords:** metabolomics, aquatic, microbial communities, post-genomic, metatranscriptomics

## Abstract

Culture-independent and *meta*-omics sequencing methods have shed considerable light on the so-called “microbial dark matter” of Earth’s environmental microbiome, improving our understanding of phylogeny, the tree of life, and the vast functional diversity of microorganisms. This influx of sequence data has led to refined and reimagined hypotheses about the role and importance of microbial biomass, that paradoxically, sequencing approaches alone are unable to effectively test. Post-genomic approaches such as metabolomics are providing more sensitive and insightful data to unravel the fundamental operations and intricacies of microbial communities within aquatic systems. We assert that the implementation of integrated post-genomic approaches, specifically metabolomics and metatranscriptomics, is the new frontier of environmental microbiology and ecology, expanding conventional assessments toward a holistic systems biology understanding. Progressing beyond siloed phylogenetic assessments and cataloging of metabolites, toward integrated analysis of expression (metatranscriptomics) and activity (metabolomics) is the most effective approach to provide true insight into microbial contributions toward local and global ecosystem functions. This data in turn creates opportunity for improved regulatory guidelines, biomarker discovery and better integration of modeling frameworks. To that end, critical aquatic environmental issues related to climate change, such as ocean warming and acidification, contamination mitigation, and macro-organism health have reasonable opportunity of being addressed through such an integrative approach. Lastly, we argue that the “post-genomics” paradigm is well served to proactively address the systemic technical issues experienced throughout the genomics revolution and focus on collaborative assessment of field-wide experimental standards of sampling, bioinformatics and statistical treatments.

## Introduction

Aquatic environments consist of a complex consortium of organisms engaged in syntrophic activities that have consequential impacts on ecosystem health and sustainability. From natural lakes and rivers, to mining impacted waters, to urban storm drains, the range of aquatic systems are dynamic and complex, which has been made increasingly evident with implementation of omics approaches. Aquatic microorganisms are responsible for the cycling of vast pools of organic materials that make their way from land to water, and are the base of all complex aquatic foodwebs, which make them of integral importance to global ecosystem stability. While invaluable insights have been gained into the collective diversity and potential function of the microbial microbiome in aquatic ecosystems worldwide, the now “standard” omics approaches of metataxonomics (microbial community structure) and metagenomics (inclusive of microbial community-wide genomics; metagenome-assembled genomes etc.), are beginning to be augmented and even replaced by “post-genomic” techniques, and for good reason (Beale et al., 2017). For instance, metatranscriptomics, which directly measures the gene expression of entire microbial communities, has provided novel insights into actual microbial activity and functionality in various aquatic environments (Lamendella et al., 2014; Falk et al., 2019; Bergsveinson et al., 2020; Reid et al., 2020). While this type of data presents us with an understanding of differential gene expression and “shifts” in activities undertaken by a community at a given time, further enhancement of these studies with metabolomics–the characterization of the low-weight molecular compounds, substrates, and enzymatic by-products within a sample matrix–that cumulatively allow for potential linkages between organism(s) genetic transcription, metabolic output, and the metabolite signature of a given environment. Thus, applying a post-genomic and/or systems-based approach (modeling of interactions within an ecosystem) to unravel the complexities of issues ranging from bioremediation to climate change research is the only means toward the ultimate end of deciphering the interrelationship of microbes and the environment and the complex biogeochemical processes which these relationships govern.

## The Answer to “Who Is There and What *Might* They Be Doing?” Does Not Serve Systemic Problems

Despite the demonstrated successes and validity of genomics across diverse fields, the burgeoning development of post-genomic approaches have underscored that distinct limitations exist on the conclusions that can be drawn from solely collecting taxonomic and/or genomic data where a mechanistic, process-based understanding of an ecosystem is required (Poretsky et al., 2014; Grossart et al., 2020). The “collective genome” uncovered via metataxonomics and metageomic studies can provide valuable insight when appropriate, though the information gathered effectively describes the genetic diversity or capacity determined by past environmental conditions - i.e., those selective pressures which allowed for a particular gene profile to arise - as opposed to the genetic capacity required to respond to contemporaneous circumstances. Proof-positive of the limitations of genomics is the frequent call for “further analysis” to identify functionally relevant organisms, and/or their actual metabolic activity as a conclusion to survey studies. Specifically, we note that the particular dynamic nature of aquatic systems–complex hydrology, chemical fluxes across interfaces, diurnal fluctuations–drives the need to mechanistically understand such processes in an *active*, not *predictive* capacity. While taxonomic identification and genomic potential may be essential in, for example, clinical pathogen research or hypothesis development, this is logically not the case in environmental studies where spatiotemporal dynamics of a community or effects of environmental stimuli on functional processes are in question. When one further considers that environmental bacteria can effectively be viewed as shuttles or capsules of genes acquired through horizontal gene transfer or mutation, instead of distinct organisms with long hereditary life cycles and genetic lineages, it is evident that environmental microbial function is more than the sum of all described constituent parts. Without analysis of community activity and outputs (metabolites and/or protein products), relying on community membership or gene content to understand larger ecosystem mechanisms is akin to trying to reassemble a deconstructed engine without a manual, and numerous miscellaneous parts. Who can be sure what parts contribute to the proper functioning of the engine, without having insight into the need, function, or output of each part?

## No Omics Is an Island

Despite warnings of technical complexities and higher investment costs, the broader environmental field has been moving rapidly away from metataxonomic surveys. Every implementation of a new “omics” is heralded as unprecedented and a new “turn-key” strategy, set to eliminate previous issues. The introduction of shotgun metagenomic sequencing presented the first opportunity to uncover the genetic potential and putative physiological traits of microbial consortia in a comprehensive and un-directed manner, and indeed have provided insight into the aforementioned “collective genome” of microbial communities across the spectrum of the Earth’s environments (Tyson et al., 2004; An et al., 2013; Cao et al., 2020). When it was begrudgingly concluded that metagenomics still did not resolve interrelationships, an eventual partnership between metagenomics and metatranscriptomics sought to improve insight into the extent of microbial genes being actively transcribed (Wu et al., 2013; Urich et al., 2014; Rowe et al., 2017). While these two approaches have together revealed an impressive amount of diversity and redundancy (a postulated genetic “fail-safe” feature) in gene sequences, and highlighted keystone species driving specific metabolic activities, studies have also demonstrated the sometimes-incompatible link between gene abundance and actual transcript activity (Bowen et al., 2014; Fraser et al., 2015; Salazar et al., 2019). While biological evolution is not a directed process (i.e., toward a unified goal), the evolution of omics approaches has clearly been toward increasing resolution of *how* a community functions, revealing, in the process, the limitation of each next-generation methodology, and the potential for biased outcomes should only one approach have been implemented.

**FIGURE 1:**
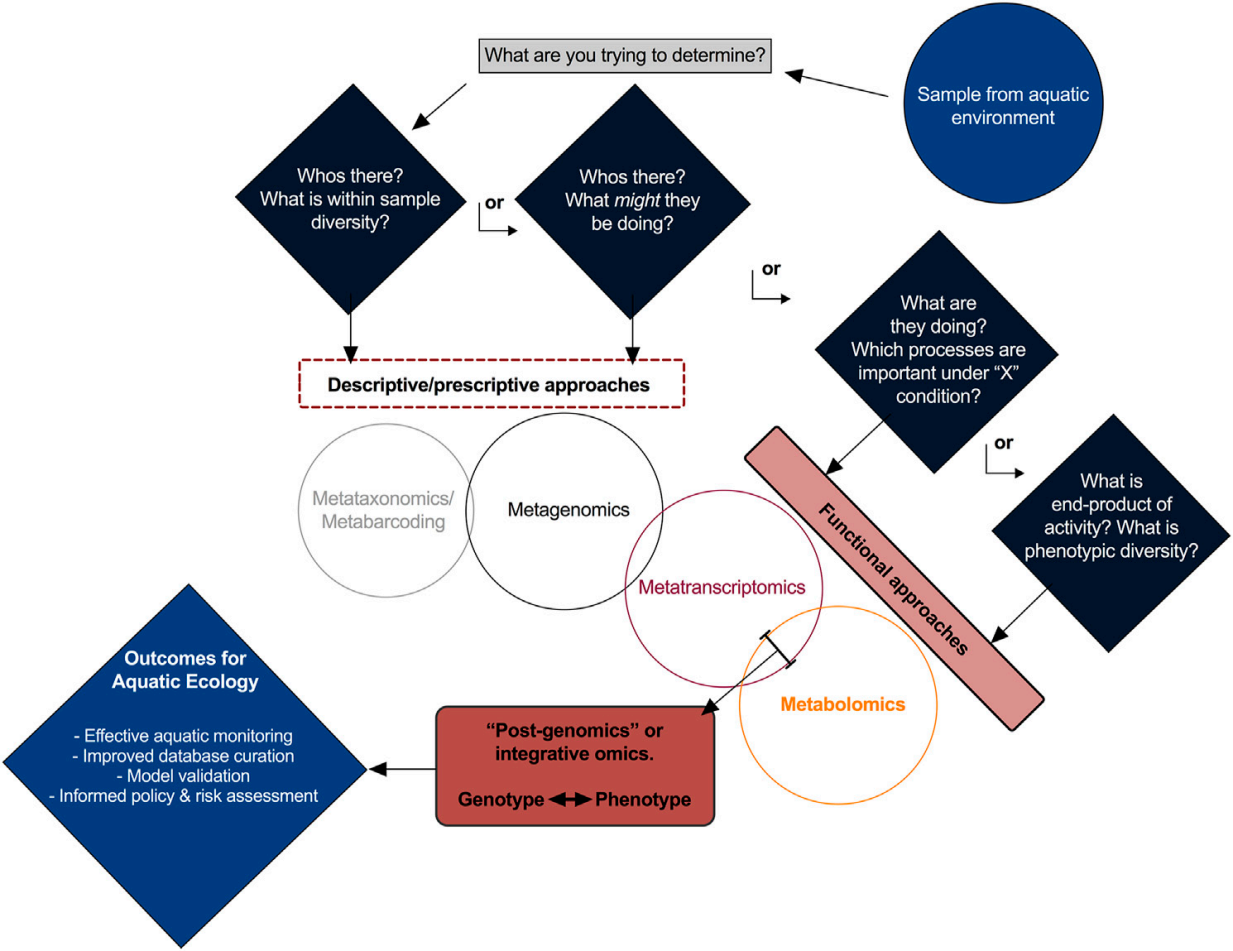
A schematic of the multi-omics approaches discussed, with corresponding scientific questions.

While the choice of omics approach depends on each unique study hypothesis (and perhaps budgetary considerations), there is merit and validity in any instance to the integration of multiple methodologies, perhaps most effectively exemplified with the joint metatranscriptomics–metabolomics approach (Figure 1). Metabolomics is proposed as the favored approach given that ongoing challenges particularly with metaproteomics studies (i.e. intricate workflows, annotation and spectra interpretation) suggest that it’s suitability particularly in aquatic environmental matrices (i.e. complex mixtures of compounds) cannot yet be fully realized until these challenges are improved upon. Further, as aquatic studies are often concerned with the characterization of relative environmental states (i.e., contaminant impact assessments or diseased vs. healthy conditions), surveying the protein profile of different environments may need to be particularly granular in order to be insightful. While all methodologies have inherent drawbacks, history has demonstrated that siloed insights gained from singular approaches will tend to force unnecessary assumptions, often resulting in more questions than answers. Though integration of metatranscriptomics and metabolomics is gaining traction, considerable challenges exist that necessitate pro-active and collective contributions from the aquatic research community.

## Integration Challenges: Acceptable or Insurmountable?

Isolated use of metatranscriptomics and metabolomics can be daunting unto themselves, let alone when applied to augment the other. Though technological advancements and refined bioinformatics approaches have made such methodologies increasingly feasible, it is worth noting that there remain considerable challenges to the tandem-application approach. These begin with the unavoidable biases inherent in the numerous sampling and preservation protocols, choice of nucleic acid isolation methods, and parameters of quality control and data filtering required during downstream bioinformatics analyses (Morgan et al., 2010; Brooks 2016; Pollock et al., 2018). Adding to this technical morass, is the array of environmental physicochemical factors influencing microbial community functionality and observed variability, especially in aquatic systems, which include water depth, temperature, sunlight exposure and seasonality, to list only few. While any “meta-omics” researcher must be cognizant of these inherent pitfalls, it must not be forgotten that these variables have long been informally deemed by the genomics field at large as “unavoidable and acceptable” so long as consistency is maintained throughout the study (Pollock et al., 2018).

The ability to learn from the technical challenges of the genomics and/or sequencing revolution is among the strongest advantages that the post-genomic research field currently has. General standardized genomics workflows, from sample acquisition to bioinformatics and appropriate statistical testing, remains a significant ongoing challenge for the cohesiveness and reproducibility of genomics, especially downstream from conventional quality control and filtering (Aguiar-Pulido et al., 2016). The unavoidable lesson to learn is the need for collective assessment and refinement of standardized integrative analysis methodologies *now,* particularly with respect to bioinformatic and correlative statistical approaches. For example, just as Young and Alfaro (2018) provided a much-needed “primer” for aquaculture researchers delving into metabolomics research, the broader field of aquatic ecology would similarly benefit from such an understanding of omics approaches, prior to wading further into the post-genomic era of environmental science. Collaborative development of harmonized analysis strategies through workshops, publication of data analyzed in parallel by different statistical means, and experimental validation of results, are all vital means for validating approaches immediately. Though methodology will naturally evolve with time, a focused development framework will also allow for methodology uptake across the broader research community.

## Discussion

While there are pressing reasons for research to expand beyond metataxonomic surveys, there is no denying the benefit they have afforded researchers worldwide to interrogate nearly every niche environment on Earth. Ease of accessibility into the field, continual cost reductions, and relatively minimal computational power for analysis continues to ensure that this approach will remain useful and dominate as a general assessment and survey tool. However, these studies are akin to fitting together one possible, albeit potentially intricate, configuration of an engine and lacking the ability to test if the engine will indeed run as anticipated. The significance of aquatic environments for global ecological and climate health demands that research activities adequately describe how the proverbial engine of these ecosystems operate.

The natural technical progression to functional omics approaches fills much of the void in a mechanistic, systems biology understanding of aquatic ecosystem dynamics. The power of functional assessment is now evident across several fields of investigation. One such pressing and increasingly emerging issue in aquatic research involves the environmental contribution to antimicrobial resistance (AMR) transmission. This environmentally inclusive view of the threat of AMR, and the integral role of the natural environment in human health, is described under the One Health paradigm supported by multiple global health agencies (American Medical Veterinary Association, 2008) and requires the concerted global efforts of clinical, biological and ecological research. To date, aquatic environments associated with potential AMR-selection point sources such as wastewater treatment plants and/or industrial or agricultural effluents have been of primary focus (Karkman et al., 2020; Mortimer et al., 2020; WHO 2020). Metagenomics has been recently employed to demonstrate that aquatic environments remain an area of AMR selection and source for further transmission via wildlife activity (Gaeta et al., 2020; Karkman et al., 2020; Mortimer et al., 2020). However, there is limited understanding of the natural or baseline resistome - the collection of bacterial resistance genes - in non-contaminated ecosystems, and in particular, aquatic environments, where dilution presumably limits human risk exposure. There is urgent need for further study given expected climate change-induced perturbations to global ecosystems (Meyer et al., 1999), and the ecologically and clinically relevant phenomenon of co-selection of AMR with non-antibiotic compounds (i.e., metals, biocides etc.) within the natural environment (Singer et al., 2016).

With permafrost thaw in the arctic creating increasingly dynamic linkages between terrestrial and aquatic ecosystems, and the likelihood of both cryogenic preservation of genetic material and microbial dormancy in sub-zero conditions, it is of vital importance to identify the functional activity of re-emergent microbes (Graham et al., 2012; Song et al., 2020; Xue et al., 2020). Initial metagenomics studies profiling the taxa and AMR profile of permafrost-associated soils have revealed a large proportion of plasmid-borne antibiotic resistance genes (AMG) relative to non-permafrost soils. This has prompted speculation that local permafrost conditions affect the composition of the resistome and selection for AMG mobility, and that as thawing dynamics (i.e., thaw slumping into adjacent waters, increased element mobility, dissolution etc.) impact microbial community structure, the transmission of AMGs will similarly be affected (Haan and Drown, 2021). While these initial findings are of importance, the impact will not be confirmed until metatranscriptomic analysis confirms plasmid mobility and/or ARG activity, and metabolomics or proteomics confirms alteration in community functional profile. The threat of emergent pathogens is but one consequence of climate change that will continue to benefit from integrative omics, as secondary impacts such as coral bleaching, species endangerment and ocean acidification require assessment of both microbial community genetic capacity and activity.

Indeed, this call was sounded nearly a decade ago by Anderson et al. (2012) which noted the advancing omics fields as integral to the study of harmful algal bloom (HAB) science (Anderson et al., 2012). While climate change and eutrophication in the world’s oceans and lakes has led to increased frequency and severity of HABs, understanding causal effects and inter-organism connectivity with respect to nutrient cycling, toxin production and ecological impact necessitates multiple omics approaches. Recently, research into HABs has utilized a joint amplicon/metabolomics approach to unravel the dynamics of marine HABs in the Gulf of Florida, wherein corresponding changes to the microbiome and metabolome were uncovered as well as implications of bioactive molecules and the potential microbial-chemical interplay during bloom events (Patin et al., 2020). Similar approaches have been used to benefit investigation of the complex coral reef microbiome, given that mechanistic understanding of aquatic holobionts is of critical importance in determining the implications their health or pathology have on the surrounding aquatic health (Wegley Kelly et al., 2018; Weber et al., 2020). Coral reefs have long been known to have a complex immune system and inflammation response that can be triggered due to any number of external disruptions, including as the result of a shift in the profile of its constituent organisms (i.e., metazoans, viruses, microorganisms). However, the intricacies and diagnostic hallmarks of coral reef immune responses, and the impact of reefs on higher trophic levels through carbon pathway alterations, cannot be well understood without the implementation of metatranscriptomics and metabolomics, as has been demonstrated through multiple studies (Quinn et al., 2016; Wegley Kelly et al., 2018; Lohr et al., 2019; Weber et al., 2020). While these larger systemic questions are obviously amenable to more integrative technologies, it is worth noting that these same approaches are equally informative for investigating more granular and/or single species dynamics. For instance, researchers investigated a bacterial consortium associated with a globally distributed diatom and found that a *Sulfitobacter* species promotes diatom cell division via secretion of the hormone indole-3-acetic acid, which serves as a signaling molecule. The potential prevalence of this mode of signaling in the oceans is corroborated by metabolite and metatranscriptome analyses that show widespread indole-3-acetic acid production by *Sulfitobacter*-related bacteria (Amin et al., 2015).

In environments where physiochemical interactions are significant determinants of microbial and/or organismal interplay, such as acid mine drainage (AMD), utilization of multiple omics approaches (alongside sensitive microscopy and imaging techniques in some cases) have been utilized for deciphering microbe-mineral interactions and the ecological impact on receiving aquatic bodies. For example, noting limitations with genomic approaches alone, Chen et al., (2015) used an integrated metagenomic-metatranscripomic approach to understand transcriptional response and adaptation mechanisms in acid mine drainage (AMD) locations. Their results indicated close linkages between transcriptional profiles and environmental conditions, with unique adaptation strategies to low-pH conditions (Chen et al., 2015). Further, metaproteomics and metabolomics have been implemented to analyze acute and chronically oil contaminated sites within the Gulf of Mexico (Kimes et al., 2013) and the Mediterranean Sea (Bargiela et al., 2015). Though many of the compounds remained unambiguously defined, integration of these approaches did reveal a network of interactive processes related to anaerobic activity and biodegradation in the presence of petroleum compounds (Bargiela et al., 2015). Overall, insights into microbial function and resilience in low-pH environments or complex hydrocarbon impacted zones have implications for predictive models of aquatic ecosystem health and sustainability, but also significant potential for resource management and biotechnological advancements such as the discovery of novel compounds (Reverter et al., 2020), as well as biomarker discovery (Pomfret et al., 2019).

In many fields, such as those discussed above, the call for a shift toward post-genomics or “multi-omics” approaches has been expressed for several years and in some cases have been embraced by at least segments of the research community (Anderson et al., 2012; Llewellyn et al., 2015; Cocolin et al., 2018; Reverter et al., 2020). While these studies tout the numerous advantages for applying them in “complex” circumstances, they are also unified in expressing concern for the equally “complex” technical challenges. Metaproteomics and metabolomics for example, can suffer from limited ability to detect low-abundance chemical species and can be prone to a high degree of biological and technical noise (among other challenges). This can be further exacerbated by still-inflated costs per sample, often limiting researchers to a lower number of biological replicates (Chen et al., 2017). Further, a single technique has yet to describe the complete metabolome of a sample, so coupling different high-resolution mass spectrometry technologies is theoretically required to describe extensive metabolomic footprints/fingerprints and enable detection of novel compounds present in very low concentration from complex mixtures (White et al., 2017). In turn, metatranscriptomics (though in itself not without technical and data analysis challenges) coupled with metabolomics have considerable issues related to cohesive statistical methods employed to identify differentially abundant transcripts and metabolites, perform correlation of these elements (more universally known as network analysis), and ultimately perform appropriate visualization and validation (Chen et al., 2017). Encouragingly however, alongside technological advancements that increase researcher accessibility into these fields, data analysis software have been developed and refined over the past decade, such as MetaboAnalyst (Chong et al., 2019). This package allows a comprehensive approach to metabolomic data analysis (integrated topology, biomarker and metabolic analyses), while also allowing compatibility with other omics datasets. These types of user-friendly analysis packages allow researchers to dive into the complexities of the post-genomic world, without need to “reinvent the wheel”.

The broader field of aquatic environmental science is clearly facing the paradigm shift toward post-genomic approaches, already revealing the potential to contribute to regulatory and policy decisions. These datasets have the not over-promized ability to improved sensitivity of biomarker selection (Pomfret et al., 2019), aid in earlier and more precise detection and modeling of ecosystem perturbations (Reid et al., 2020; Hipsey et al., 2020), and improved risk assessment frameworks that includes functional characteristics of microbial behavior (Cocolin et al., 2018). This type of data also stands to revolutionize the time and expense-consuming (and often-neglected) pursuit of microbial cultivation that is required for experimental and ecological validation of gene annotations and activities. As metabolic capabilities are linked to the transcriptional activities of specific organisms within the environment via these multi-omics datasets, it can also be expected that some previously uncultured species might be possible to cultivate or co-cultivate with the recreation of required metabolic conditions (Gutleben et al., 2018).

While the healthy skeptic is correct to not view any analysis paradigm as a “holy grail” means or end, the potential power of these integrated approaches far outweighs the inherent issues. Application of metatranscriptomics and metabolomics, through careful analytical and statistical frameworks, can and will enable identification of functional trait information, annotation of hypothetical proteins via their association with known metabolites, and expansion and curation of current and novel databases. To this end, mechanistic understanding of the genetic expression linkages to metabolic endpoints of aquatic microbial communities serves as the necessary starting point at which to build the predictive capacity of systems biology.

## Data Availability

The original contributions presented in the study are included in the article/Supplementary Material, further inquiries can be directed to the corresponding authors.
